# A rapid, early detection of oral squamous cell carcinoma: Real time PCR based detection of tetranectin

**DOI:** 10.22099/mbrc.2019.31544.1365

**Published:** 2019-03

**Authors:** Teena Sajan, Smitha Murthy, Rijesh Krishnankutty, Joyeeta Mitra

**Affiliations:** Credora Life Sciences, Horamavu, Bangalore, Karnataka-560043, India

**Keywords:** Tetranectin, OSCC, Biomarker, Real Time PCR

## Abstract

The current study is focused on determining the mRNA expression levels of tetranectin, to detect oral squamous cell carcinoma (OSCC) and thus aiding in its classification at an early stage. RNA was isolated and cDNA synthesis was performed from the saliva samples of the patients and healthy individuals. A semiquantitative PCR based analysis was performed prior to quantitative and expression based analysis using Real time PCR. The study showed that the mRNA levels are much lesser in patients suffering from dysplastic and metastatic tumors as compared to healthy individuals (P≤0.05). This study can be a breakthrough in medical and dentistry studies. One of the most common malignant carcinomas, OSCC is a type of cancer of the mouth. Though surgical methods have been quite effective in delaying the metastasis, the detection methods using histology parameters are not very efficient and the disease is diagnosed generally in the last stages of the cancer. Tetranectin is a protein biomarker which has been used for detection of several cancers including oral cancer where the protein quantity is calculated.

## INTRODUCTION

Oral squamous cell carcinoma (OSCC) is a very common human cancer with a very high annual incidence of 300,000 new cases worldwide [1, 2]. The crucial factor for patient survival rates is the metastasis to the lymph nodes. A detectable lymph node involvement is noticed in 50% of patients with OSCC and less than 40% of patients presenting with lymph node metastasis have a survival rate of less than five years [3, 4]. The treatment method involves a surgical procedure of Radical neck dissection (RND) to remove a portion of the neck. The tissues are histologically examined to clinically diagnose individuals with lymph node metastasis. However, this procedure is not always accurate as 10-20% of individuals clinically diagnosed with lymph node metastasis turn out to be negative [5, 6]. The clinical diagnosis of metastasis free status is less accurate as nearly one third of these individuals have a positive lymph node metastasis upon postoperative histological examination [7]. The low sensitivity of detecting nodal metastasis by the routine elective neck dissection (END) and the pathological examination of the removed lymph nodes are not appropriate as individuals with lymph node metastasis are falsely diagnosed as non-metastatic and the procedure is not necessary for individuals diagnosed as non-metastatic. The procedure causes long term discomfort, disfigurement, pain in the shoulder and neck disability and hence is not recommended for non-metastatic patients, leaving a certain percentage of patients untreated who have been mistakenly diagnosed negative for the case [8, 9]. The challenge at hand is to ensure optimal management and accuracy in staging of lymph node metastasis; the current preoperative methods are suboptimal and misdiagnose the presence or absence of metastasis in many patients. Cancer biomarkers can be helpful for the prediction or detection of lymph node metastasis in individuals with OSCC [10].

Detection of oral cancer from saliva can be of benefit as it has direct contact with the cancer lesions, the saliva is an intricate oral fluid composed of various secretions from the salivary gland and the gingival crevicular fluid. It is of interest to use saliva as a diagnostic tool as it can be obtained through noninvasive method and helps in monitoring a wide range of biomolecules [11]. Significance of salivary biomarkers are widely reported; an elevated level of CA 15 and Her2/neu and low levels of p53 are reported for breast cancer [12], elevated CA 125 is reported in patients with ovarian cancer [13], salivary leptin is expressed in a higher quantity in salivary gland tumors compared to healthy parotid tissue [14]. Inflammatory cytokines have also been investigated as biomarkers [15], Interleukin 8 are reported to be higher in patients having squamous cell carcinoma of head and neck region compared to individuals without cancer [16]. It has been established that a combination of RNA markers and protein aids in increasing the detection rate of OSCC [17]. Hence it is of priority to develop a salivary diagnostic tool for the identification and classification of patients with premalignant lesions and malignant lesions [18]. 

Tetranectin is a C- type lectin domain family 3, member B (*CLEC3B*) glycoprotein in human. It has a prominent role in the regulation of fibrinolysis, proteolytic processes and tissue growth; it is present in cells including monocytes, neutrophils, fibroblasts and osteoblasts [19, 20]. Tetranectin has been investigated as a biomarker; it is down regulated in metastatic cancer like lymph node cancer, oral cancer, colonic cancer, ovarian cancer and breast cancer [21, 22]. It also has a role in neurological disorders like Parkinson disease, multiple sclerosis, epilepsy and Alzheimer’s disease [23, 24]. Tetranectin has also been reported to play a vital role in coronary artery diseases; the regulation of fibrinolysis and proteolytic procedures is by tetranectin and abnormal changes in the level can have an impact on coagulation and fibrinolysis during coronary artery disease progression [25, 26]. Tetranectin has been reported as a potential biomarker for OSCC due to the significant reduction in the levels in metastatic OSCC compared to primary OSCC, this is in respect with both serum samples and saliva samples [27]. The decreased levels of tetranectin were associated with cancer progression; in ovarian cancer the decreased levels of plasma tetranectin is an indication of adverse prognosis than the cancer stage progression [28, 29]. In metastatic breast cancer patients the low levels of serum tetranectin correlates to poor response of the patient to the treatment methods [30, 31]. Hence the use of tetranectin as a potential biomarker for metastasis and clinical classification of patients with solid and metastatic tumors can aid in the prognosis, diagnosis and treatment options for the patients.

## MATERIALS AND METHODS

**Sample Collection:** Clinical samples were retrieved from the Dental Hospitals from Bangalore. Saliva samples of 15 healthy volunteers, 15 dysplasia patients and 15 metastatic OSCC patients were collected. The classification of dysplasia and cancer cases was based on the TNM classification of carcinomas of the oral cavity with respect to their histological report.

**Extraction of RNA: **The plastic wares and glass wares used were DEPC treated. The saliva samples were centrifuged at 10,000 rpm for 10 minutes, to the pellet 1ml of Trizol Reagent (Takara Bio Inc, Japan) was added, mixed by grinding, and incubated at room temperature for 15 mins. It was then centrifuged at 10,000 rpm for 5 minutes and the supernatant was transferred to a fresh sterile microfuge tube. Equal volume of Chloroform: Isoamyl alcohol (24:1) was added, mixed gently and incubated in ice for 2 minutes. The tubes were centrifuged at 12,000 rpm for 10 minutes, the supernatant was transferred to a fresh tube and 500µl of isopropanol was added and incubated at -20°C for 1 hour. The RNA was pelleted down by centrifuging at 10,000rpm for 10 minutes. The pellet was vortexed gently with 300µl of 70% of ethanol and then centrifuged at 10,000 rpm for 10 minutes. The pellet was air dried. 20µl of RNase free water was added to the pellet and gently dissolved. The RNA was treated with DNase enzyme to remove any traces of DNA contamination. The concentration and purity of RNA was assessed using a spectrophotometer (Sartorius). A 1µL aliquot of RNA was pipetted onto the apparatus pedestal. RNA with an absorbance ratio at 260 and 280 nm (A260/A280) between 1.8 and 2.2 was indicative of pure RNA. 

**cDNA synthesis: **After quantification, RNA was reverse transcribed using oligo dT (Sigma Aldrich). 100 ng of RNA was aliquoted to a fresh sterile microfuge tube to which 2µl of oligo dT was added and incubated at 70°C for 5 minutes and immediately transferred to ice. To this 2µl of dNTPs, 1µl of Reverse Transcriptase enzyme (Biolabs, New England) and 2µl of 10x Reverse transcriptase buffer was added and made up the volume to 25µl using RNase free water. This mixture was incubated at 42°C for 90 minutes and reaction was terminated by incubating at 70^o^C for 15 minutes. The cDNA were confirmed by performing PCR for GAPDH.

**PCR Design and synthesis: **The primers for quantification analysis were designed using Perkin Elmer Primer Express® software. The Melting temperature (Tm) was calculated and the synthesized primers were purified by HPLC. The quantified cDNA was used to detect the presence of Tetranectin using the specific primers below and Primer optimization was done in a gradient PCR in range of 50-60°C. 

**PCR Amplification** **:** The quantified cDNA was used to detect the presence of Tetranectin gene using the specific primers for CLEC3B gene: Tetranectin FP-5’AGA CTG AGA TCA CCG CGC AA3’ and Tetranectin RP -5’CAC GAT CCC GAA CTG GCA GA3’. A 20µl reaction was set using following PCR components: 2μl of 10X reaction buffer with 1.5mM MgCl_2_, 2μl of 2.5mM dNTPs, 2μl each of forward and reverse primer, 0.3μl of Taq DNA polymerase, 1μl of template cDNA and the volume made upto 20µl with nuclease free water. The PCR was programmed as follows: 3 minute denaturation step at 94°C, then 40 cycles of amplification at 94°C for 1 minute, 50-60°C for 30 seconds and 72°C for 1 minute, in addition to a 10 minutes extension at 72°C.

**Gel imaging and semi quantitative analysis: **The PCR products were loaded on a 1.2% agarose gel stained with Ethidium Bromide and visualized under UV transilluminator (BioBee, India) and the image was captured under UV light. The band intensity was measured using the software GelAnalyzer2010a and was compared.

**Relative Quantification using Real Time PCR:** The quantification for Control samples was done in Applied Biosystems Step One Real Time PCR using the SYBR Green Chemistry. The reaction was carried out in a 10µl reaction volume with the following components: first strand cDNA 0.5 µL, SYBR Green Master Mix (2X) 5.0 µL, Forward primer (10 µM) 0.5 µL, Reverse primer (10 µM) 0.5 µL and Nuclease-free water 3.5 µL. All reaction components are procured from Life Technologies. Initial steps of RT-PCR was 2 minutes at 50°C for UNG erase activation, followed by a 10 minutes hold at 95°C. Cycles (n = 40) consisted of a 15 second melt at 95°C, followed by a 30 second annealing/extension at 55°C. The final step was 60°C incubation for 30 second for extension. All reactions were performed in duplicates against a serially diluted standard. Threshold cycle (Ct) analysis of all samples was set at automatic detection by the system. The benign and malignant samples were then tested against control samples using gene expression analysis. GAPDH was used as the endogenous control gene. 

**Statistical Analysis** **:** The expression levels of tetranectin were analyzed using SPSS software version 17 (SPSS Inc., Chicago, IL, USA) using one-way ANOVA, followed by post hoc comparisons using Tukey’s HSD.

## RESULTS

The concentration and purity of the RNA extracted from the saliva samples were recorded using a spectrophotometer (Sartorius Stedium). The results depict a good RNA yield with a purity of 2.0- 2.2. The cDNA synthesized was also assessed for its integrity by performing PCR with the GAPDH gene. The cDNA samples were diluted to 200ng/µl for the Tetranectin encoding CLEC3B gene PCR analysis. 

Gradient PCR was performed of the synthesized primers and the annealing temperature was finalized at 54°C giving a single, prominent band of 134bp.The quantified cDNA was used to detect the presence of CLEC3B gene (tetranectin) using the specific primers at annealing temperature of 54°C to yield a single amplified band of 134bp. 

**Figure 1:**
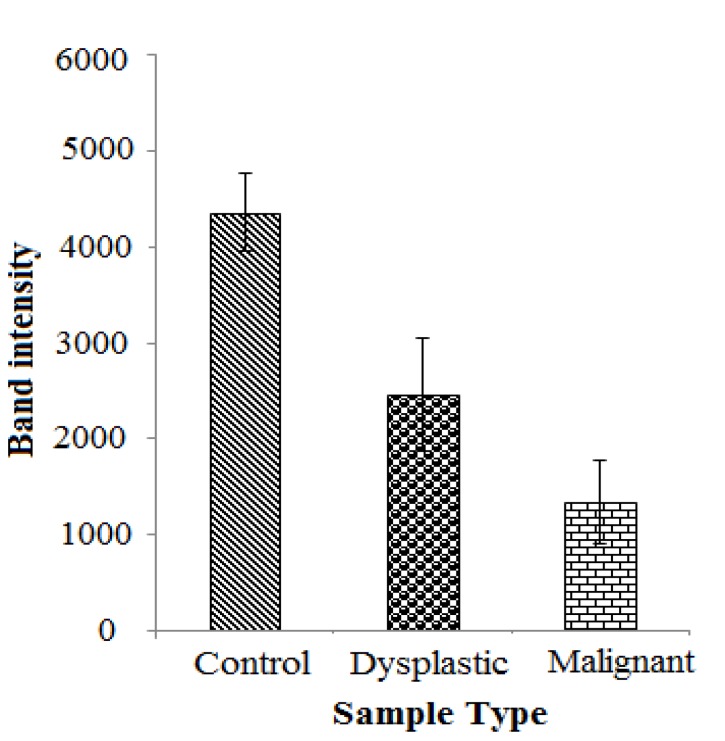
Mean expression levels of the *CLEC3B* gene (tetranectin) for the control, dysplastic and malignant samples by semi-quantitative analysis

A significant difference was seen in the semi quantitative analysis between the control samples and the OSCC samples (Fig. 1). The gene expression was significantly higher in the control samples than the dysplastic and metastatic samples (P≤0.05). The mRNA expression levels were least in the metastatic samples (P≤0.05).

**Figure 2:**
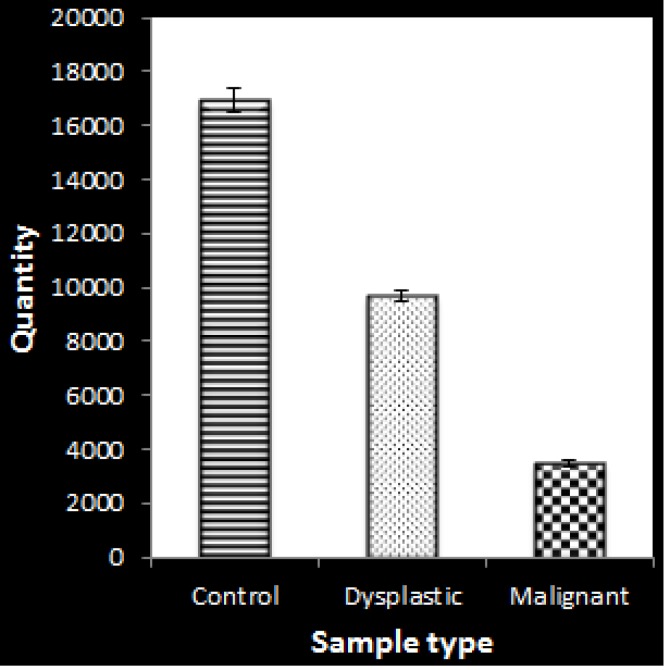
Mean Relative quantitation of each sample group has been plotted for the expression analysis of *CLEC3B* (tetranectin) by quantitative real time PCR

**Figure 3:**
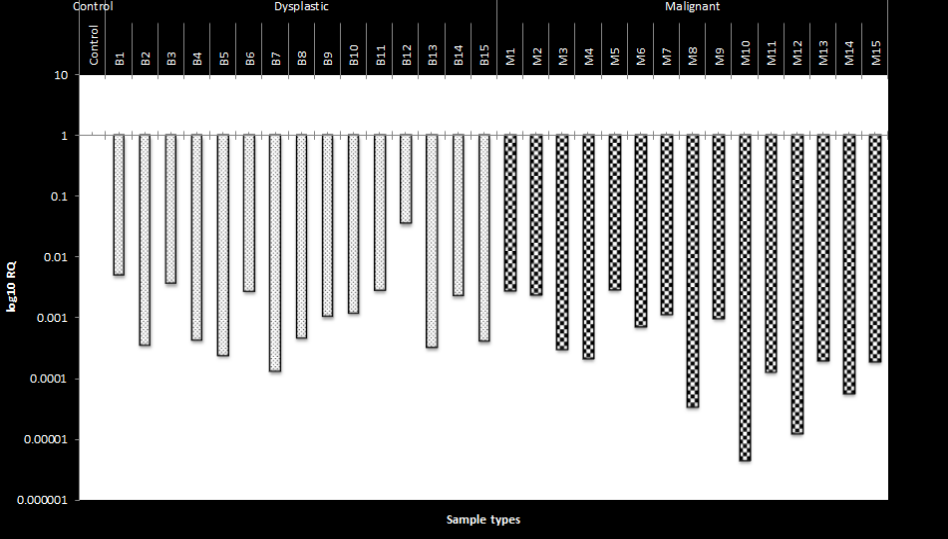
RQ log scale of the expression of tetranectin showing underexpression in the dysplastic and malignant OSCC samples

A relative quantification was done to determine the expression level of CLEC3B (teranectin) with reference standards (Fig. 2). The comparison was done between the controls samples, the dysplastic and metastatic OSCC samples to understand the expression levels. The expression was higher in the control samples (P≤0.05) and was under-expressed in the dysplactic and metastatic samples (P≤0.05) of OSCC patients as shown in (Fig. 3). Values greater than 1 is considered to be over- expressed and lesser than 1 is under- expressed.

## DISCUSSION

The main factor in the poor survival rate of OSCC patients is the delayed diagnosis. The clinical examination of biopsies, tissue staining and cytological studies has various limitations and is applicable only on a smaller patient group. The need for highly trained personnel for the examination is time consuming and expensive; since it is a surgical method and the patient distress is also to be taken into consideration. The development of a salivary biomarker for OSCC detection is a promising non- invasive approach for the detection of oral cancer. Salivary biomarkers are efficient diagnostic medium for the detection of various diseases. Diagnosis of several diseases from salivary samples using RNA have been reported, expression analysis of the salivary transcriptome between healthy and diseased conditions have been reported. This helps in the detection and classification of the progression of the disease [32].

The present study was aimed to identify tetranectin (TN) as a potential biomarker to differentiate metastatic, dysplastic and healthy individuals. The study suggests that tetranectin can be considered as a potential biomarker as it is significantly reduced in metastatic OSCC compared to dysplastic and the control group. Tetranectin is a distinct protein belonging to the C- type lectin family. The human protein is a homotrimer forming a triple alpha helical coiled structure; each monomer has a C-type lectin domain that is connected to the alpha helix [27]. Tetranectin binds to a circulating zymogen; specifically to the kringle 4 domain of plasminogen [19]. The plasminogen activation has an important role in tumor invasion and metastasis.

Tetranectin coded by CLEC3B gene is often used as a biomarker for detection of several diseases like Parkinson’s disease. Tetranectin has been used as a potential protein biomarker in diagnosing epileptic patients, wherein TN is present in the cerebrospinal fluid of the patients but not in healthy controls [23]. Tetranectin has been identified as potential biomarkers for Stable Coronary artery Disease. TN levels were higher in patients with increased number of diseased arteries. Additionally, IHC analysis showed that TN expression was significantly higher in atherosclerotic arteries as compared to healthy control tissues. Though several studies have been performed on role of tetranectin biomarkers in detecting diseases, not much information was there for its role in detecting OSCC [33]. 

The possible application of tetranectin as a suitable marker for detection of OSCC was investigated and concluded that the Tetranectin protein levels were significantly under-expressed in both serum and saliva samples of metastatic OSCC when compared to primary OSCC. Work by Almabrook et al. [34] was also performed in our Lab, Credora Life Sciences, where initial confirmation of the primer testing was performed, as also mentioned in the methodology section of the paper. In a tumor environment, tetranectin seems to be consumed for the proteolytic activity which is required for tumor metastasis, this results in lesser amount of tetranectin in patients with metastasis compared to primary tumor [21]. 

The current work was to study the expression levels of the tetranectin gene. Gene expression was studied using semi-quantitative approach and quantitative analysis by real time PCR, the comparative analysis was performed for both sets of data. The data from both analyses gave similar expression where; the healthy samples had higher expression of tetranectin when compared to OSCC patients. Also, the metastatic patients had even more under-expressed gene expression of tetranectin when compared to dysplastic samples. Hence, it could be concluded that expression of Tetranectin at genomic level can be used as a suitable biomarker for detection and diagnosis of OSCC, and also be used to detect the cancer progression.
